# The *Arabidopsis* JMJ29 Protein Controls Circadian Oscillation through Diurnal Histone Demethylation at the *CCA1* and *PRR9* Loci

**DOI:** 10.3390/genes12040529

**Published:** 2021-04-05

**Authors:** Hong Gil Lee, Pil Joon Seo

**Affiliations:** 1 Department of Chemistry, Seoul National University, Seoul 08826, Korea; hglee1@snu.ac.kr; 2 Plant Genomics and Breeding Institute, Seoul National University, Seoul 08826, Korea

**Keywords:** circadian clock, evening complex, histone methylation, JMJ29, CCA1, PRR9

## Abstract

The circadian clock matches various biological processes to diurnal environmental cycles, such as light and temperature. Accumulating evidence shows that chromatin modification is crucial for robust circadian oscillation in plants, although chromatin modifiers involved in regulating core clock gene expression have been limitedly investigated. Here, we report that the Jumonji C domain-containing histone demethylase JMJ29, which belongs to the JHDM2/KDM3 group, shapes rhythmic changes in H3K4me3 histone marks at core clock loci in *Arabidopsis*. The evening-expressed JMJ29 protein interacts with the Evening Complex (EC) component EARLY FLOWERING 3 (ELF3). The EC recruits JMJ29 to the *CCA1* and *PRR9* promoters to catalyze the H3K4me3 demethylation at the cognate loci, maintaining a low-level expression during the evening time. Together, our findings demonstrate that interaction of circadian components with chromatin-related proteins underlies diurnal fluctuation of chromatin structures to maintain circadian waveforms in plants.

## 1. Introduction

The circadian clock is an internal timekeeping mechanism that generates biological rhythms with a period of ~24 h and synchronizes plant growth and development with environmental cycles. Multiple transcriptional feedback loops establish the basic architecture of the plant circadian clock. In *Arabidopsis*, the two morning-expressed single-MYB transcription factors, CIRCADIAN CLOCK–ASSOCIATED 1 (CCA1) and LATE ELONGATED HYPOCOTYL (LHY), repress transcription of the evening-expressed *TIMING OF CAB EXPRESSION 1* (*TOC1*)/*PSEUDO RESPONSE REGULATOR 1* (*PRR1*), that in turn represses *CCA1* and *LHY* expression, forming the central loop [1,2,3,4]. The central loop is further interconnected with additional transcriptional loops. In the morning, a subset of the PRR family, including PRR7 and PRR9, associate with the *CCA1* and *LHY* promoters to repress expression [5,6], and PRR5 also binds to the *CCA1* promoter late in the day [7]. Evening-expressed clock components, such as TOC1 and the Evening Complex (EC), contribute to repressing the morning genes [8,9,10].

Additional layers of regulation add complexity to precise circadian oscillation. In particular, the intimate association between chromatin modification and the circadian clock is beginning to emerge [11]. Rhythmic expression of core clock genes is linked to the diurnal changes in histone 3 acetylation (H3ac), e.g., H3K56ac and H3K9/14ac, and histone 3 lysine 4 trimethylation (H3K4me3) levels at the gene promoters in *Arabidopsis* [11,12,13,14]. The two active histone marks have different modes of action in clock gene control: temporal H3ac deposition facilitates open chromatin formation to allow peak expression of clock genes [15,16]; whereas the H3K4me3 mark blocks access of clock repressor at the core clock gene promoters [16], shaping elaborate circadian waveforms. Despite the molecular connection between chromatin modification and the circadian control, a limited number of chromatin modifiers responsible for circadian histone modification have been demonstrated.

As for the diurnal accumulation of H3ac, HISTONE DEACETYLASE 9 (HDA9) regulates circadian oscillation by repressing *TOC1* expression. HDA9 associates with an EC component, ELF3, and the EC enables HDA9 to bind to the *TOC1* promoter, stimulating H3 deacetylation to inhibit *TOC1* expression [17]. The temporal binding of HDA9 is defined by the EC that acts at nighttime, and thus, HDA9 facilitates declining phase of *TOC1* expression during the night period [17]. A couple of HDACs also participate in circadian oscillation. PRR5, PRR7, and PRR9 interact with HDA6 and HDA19 to robustly repress *CCA1* and *LHY* [18,19]. In support, treatment with the potent HDAC inhibitor TSA promotes *CCA1* expression and possibly lengthens circadian period [19].

The H3K4me3 level at core clock genes is delicately regulated by antagonistic actions of histone methyltransferases and histone demethylases. The SET DOMAIN GROUP 2 (SDG2)/ARABIDOPSIS TRITHORAX–RELATED 3 (ATXR3) protein globally deposits the H3K4me3 mark at multiple clock-associated loci to increase gene expression [16,20]. The *SDG2*/*ATXR3*-deficient mutants exhibit a reduced H3K4me3 accumulation, increased clock repressor binding, and thereby a low-amplitude expression of core clock genes [20]. Several Jumonji C (JmjC) domain-containing histone demethylases are known to counterbalance clock gene expression. The dusk-expressed *JMJ30*/*JMJD5* gene regulates expression of *CCA1* and *LHY*, which reciprocally repress *JMJ30*/*JMJD5* [21]. Moreover, CCA1 and LHY also form a feedback relationship with *JMJ14* and *SDG2*, which facilitates rhythmic H3K4me3 dynamics for the clock genes as well as their output genes [20].

The coordinated control of H3ac and H3K4me3 accumulation has also been demonstrated in the regulation of circadian oscillation [11]. The morning-expressed CCA1 and LHY transcription repressors interact with the protein complex containing the LYSINE-SPECIFIC HISTONE DEMETHYLASE 1 HOMOLOG 1 (LDL1) and LDL2 H3K4 demethylases and the HDA6 histone deacetylase [18]. The LDL1/2-HDA6 complex is recruited to CCA1/LHY-target genes, such as *TOC1* [18]. In the morning, the *TOC1* gene is suppressed synergistically by H3 deacetylation and H3K4 demethylation. By contrast, in the evening, because *CCA1* and *LHY* are expressed at a low level, the binding of LDL1/2-HDA6 complex to the *TOC1* promoter is impaired, increasing *TOC1* expression [18]. Overall, interplays between chromatin modifiers and core circadian clock components fine-tune circadian oscillation and thereby plant growth and development.

Despite the advances in the understanding of epigenetic regulation of circadian oscillation, chromatin modifiers contributing to the rhythms of chromatin structures and their action mechanisms remain to be fully elucidated. Here, we report that JMJ29 participates in the circadian regulation of morning-expressed *CCA1* and *PRR9* genes. JMJ29 physically associates with an EC component ELF3, and both EC and JMJ29 bind to the *CCA1* and *PRR9* promoters, maintaining their expression at a low level during evening time. Our findings suggest that temporal regulation of histone methylation underlies robust circadian rhythmicity.

## 2. Materials and Methods

### 2.1. Plant Growth Conditions

The *Arabidopsis thaliana* ecotype Col-0 was used. Plants were grown under neutral day conditions (NDs; 12 h light/12 h dark cycles) with cool white fluorescence (120 μmol photons m^−2^ s^−1^) at 22–23 °C. The *jmj29-1, elf3-1, pJMJ29:JMJ29-GFP/jmj29-1*, and *pELF3:ELF3-MYC/elf3-1* mutants were previously described [22,23]. To investigate the biological function of *JMJ29*, T-DNA insertional *jmj29-1* (Salk_021597) mutant seeds were obtained from the *Arabidopsis* Biological Research Center.

### 2.2. RNA Extraction and Quantitative RT-PCR (RT-qPCR)

Total RNAs were isolated using TRI agent (TAKARA Bio, Singa, Japan). Two μg of total RNAs were pretreated with an RNAse-free DNAse and then used to synthesize first-strand cDNA using Moloney Murine Leukemia Virus (M-MLV) reverse transcriptase (Dr. protein, Seoul, South Korea) and oligo (dT18). Synthesized cDNAs were used for PCR amplification.

Quantitative RT-PCR reactions were conducted in 96-well blocks using the Step-One Plus Real-Time PCR System (Applied Biosystems, Foster City, CA, USA). The PCR primer sequences used in this study are listed in Supplementary Table S1. *EUKARYOTIC TRANSLATION INITIATION FACTOR 4A1* (*eIF4A*) gene (At3g13920) was used as a reference gene for relative normalization. RT-qPCR was performed with SYBR Master Mix (Enzynomics, Seoul, South Korea) using the △△C_t_ method. The specificity of the RT-qPCR reactions was determined by melt curve analysis of the amplified products using the standard method installed in the system.

### 2.3. Yeast Two Hybrid (Y2H) Assays

The BD Matchmaker system (Clontech, Mountain View, CA, USA) was employed to conduct Y2H assays. The pGADT7 vector was used for the GAL4 activation domain (AD) fusion, and the pGBKT7 vector was used for GAL4 binding domain (BD) fusion. PCR products were subcloned into the pGBKT7 and pGADT7 vectors. The recombinant expression constructs were co-transformed into yeast pJG69-4A cells harboring the *LacZ* and *His* reporter genes, and transformed cells were selected by incubating on SD/-Leu/-Trp medium and SD/-Leu/-Trp/-His/-Ade. 

### 2.4. Bimolecular fluorescence Complementation (BiFC) Assays

The full-size *ELF3*, *ELF4*, and *LUX* coding sequences were fused in-frame to the 5′ end of a gene sequence encoding the N-terminal half of EYFP in the pSATN-nEYFP-C1 vector (E3081). The *JMJ29* gene was fused to the 3′ end of a gene sequence encoding the C-terminal half of EYFP in the pSATN-cEYFP-N1 vector (E3084).

For protoplast isolation, 14-day-old seedlings grown under the ND conditions were harvested in 20 mL 0.5 M mannitol solution (90Φ plate) and incubated for 1 h at room temperature (RT). Then, the 0.5 M mannitol solution was replaced with 20 mL enzyme solution (2% Viscozyme L, 1% Celluclast 1.5 L, 1% Pectinex Ultra SP-L in MMC, adjusted to pH 5.8 by NaOH and sterilized through a 0.2 μm syringe filtering) and incubated in the dark for 12–14 h at RT. The protoplasts were collected by centrifugation at 100 *g* for 7 min and washed twice with the W5 solution (0.1% glucose, 0.08% KCl, 0.9% NaCl, 1.84% CaCl_2_, and 2 mM MES, adjusted to pH 5.7). For BiFC assays, 10 μg nYFP- and 10 μg cYFP-fusion plasmid DNAs were used to transfect 1.2 × 10^6^ protoplasts in 300 μL MMg solution (4 mM MES containing 0.4 M D-mannitol and 15 mM MgCl_2,_ adjusted to pH 5.7 with 2 M NaOH). The recombinant plasmids were co-transfected with a nuclear marker (mCherry-NLS plasmid). After 14 h incubation, the protoplasts were harvested and observed using Confocal Quantitative Image Cytometer CQ1 (YOKOGAWA).

### 2.5. Co-Immunoprecipitation Assays

*35S:ELF3-HA* and *35S:JMJ29-GFP* constructs were co-transfected into protoplasts isolated from 14-day-old *Arabidopsis* seedlings, and the transfected protoplasts were incubated in the dark for 16 h at RT. The protoplasts were collected by centrifugation at 300 *g* for 5 min. About 2 × 10^6^ protoplasts were homogenized in IP buffer (25 mM Tris-HCl, 150 mM NaCl, 0.5 % Triton X-100, 1 mM EDTA, and 1 % protease inhibitor, adjusted to pH 7.5), incubated for 30 min on ice, and centrifuged at 15,000 *g* for 10 min at 4 °C to collect supernatant. Then, 50 μL of Protein A/G Agarose Beads (SC-2003, Santa Cruz Biotechnology, Santa Cruz, CA, USA) were added, and the mixture was incubated for 3 h at 4 °C with gentle shaking (50 rpm). The beads were removed by centrifugation at 14,000 *g* for 5 min at 4 °C, and antibodies were then added to the supernatant. After overnight incubation at 4 °C, 50 μL of protein A-Sepharose (P9424, Sigma-Aldrich, USA) was added and incubated for an additional 6 h at 4 °C. The beads were collected by centrifugation at 100 *g* for 3 min at 4 °C and washed five times with IP buffer. The proteins were eluted by boiling in SDS-PAGE sample buffer for 5 min and subjected to western blot analysis. The anti-HA (ab9110, Abcam, Cambridge, UK) and anti-GFP (ab290, Abcam, Cambridge, UK) were used for immunoprecipitation and western blot analyses.

### 2.6. Chromatin Immunoprecipitation (ChIP) Assays

The *pJMJ29:JMJ29-GFP/jmj29-1*, *pELF3:ELF3-MYC/elf3-1*, *jmj29-1*, and *elf3-1* plants [22,23] were used for ChIP assays. Plant materials used for the experiments were grown under NDs for 2 weeks. Harvested plant materials were fixed in 1% formaldehyde for 20 min with vacuum infiltration and ground in liquid nitrogen. Chromatin solubilized by the nuclei lysis buffer (50 mM Tris-HCl pH 8.0, 10 mM EDTA pH 8.0, 1% SDS, 1 mM PMSF, and 1× PIs) was sonicated at 4 °C to generate ~500 bp fragments using a Bioruptor Pico (Diagenode). The chromatin solutions were ultrasonicated for 10 cycles (30 s ON and 30 s OFF for each cycle on full power). The antibodies, including anti-GFP (ab290, Abcam, Cambridge, UK), anti-MYC (05-724, Millipore, Billerica, USA), anti-H3 (04-928, Millipore, Billerica, MA, USA), anti-H3K4me3 (07-473, Millipore, Billerica, USA), and anti-H3K9me2 (ab1220, Abcam, Cambridge. UK), and agarose A/G beads (SC-2003, Santa Cruz Biotechnology, Santa Cruz, CA, USA) were used for ChIP. The fragmented DNAs were purified using a DNA elution kit. Precipitated DNA level was quantified by quantitative real-time PCR using specific primer sets listed in Supplementary Table S2. ChIP-qPCR values were normalized as a percentage of the input DNA.

### 2.7. Transient Gene Expression Assays

For transient expression assays using *Arabidopsis* protoplasts, reporter and effector plasmids were employed [17,24,25]. The reporter plasmid contained a minimal 35S promoter sequence as well as the *GUS* gene [26]. The core elements on the *CCA1* and *PRR9* promoters were subcloned into the reporter plasmid. To construct the *p35S:JMJ29*, *p35S:ELF3*, *p35S:ELF4*, and *p35S:LUX* effector plasmids, coding regions were subcloned into the effector vector containing the CaMV 35S promoter. Recombinant reporter and effector plasmids were co-expressed into *Arabidopsis* protoplasts by a polyethylene glycol (PEG) method [27]. GUS activities were measured based on the fluorometric method. A CaMV 35S promoter-luciferase construct was also co-transformed as an internal control to normalize GUS activity. The luciferase assay was performed using the D-luciferin (BT11-1000Na, BioThema, Handen, Sweden).

### 2.8. Bioluminescence Assays

Seeds were sown in 1/2 MS-solid medium and stratified in darkness at 4 °C for 3 days, and germinated seedlings were entrained for 3 weeks under ND conditions at 22–23 °C. To examine circadian oscillations in protoplasts, mesophyll protoplasts were isolated from the lower epidermal layer of 3-week-old *Arabidopsis* seedlings. Whole seedlings were soaked in 10 mL of an enzyme solution (400 mM mannitol, 20 mM KCl, 20 mM MES-KOH [pH 5.7], 10 mM CaCl_2_, 1% Cellulase R10, 0.5% Macerozyme R10, and 0.1% bovine serum albumin) for 16 h in the dark. The isolated protoplasts were filtered through sterile 100 mm stainless mesh and resuspended in W5 solution. The *pCCA1:LUC* and *p35S:ELF3* plasmids were prepared by PEG purification. Then, the recombinant constructs were transiently introduced into *Arabidopsis* protoplasts via PEG-mediated transformation [23].

Luminescence rhythms were monitored using the Tristar2 LB 942 Multimode Microplate Reader (Berthold Technologies, Wildbad, Germany). The circadian period was estimated using the Fast Fourier Transform-Nonlinear Least Squares (FFT-NLLS) suite of programs available in the Biodare2 software.

## 3. Results

### 3.1. Altered Circadian Oscillation in jmj29 Mutant

**Figure 1:**
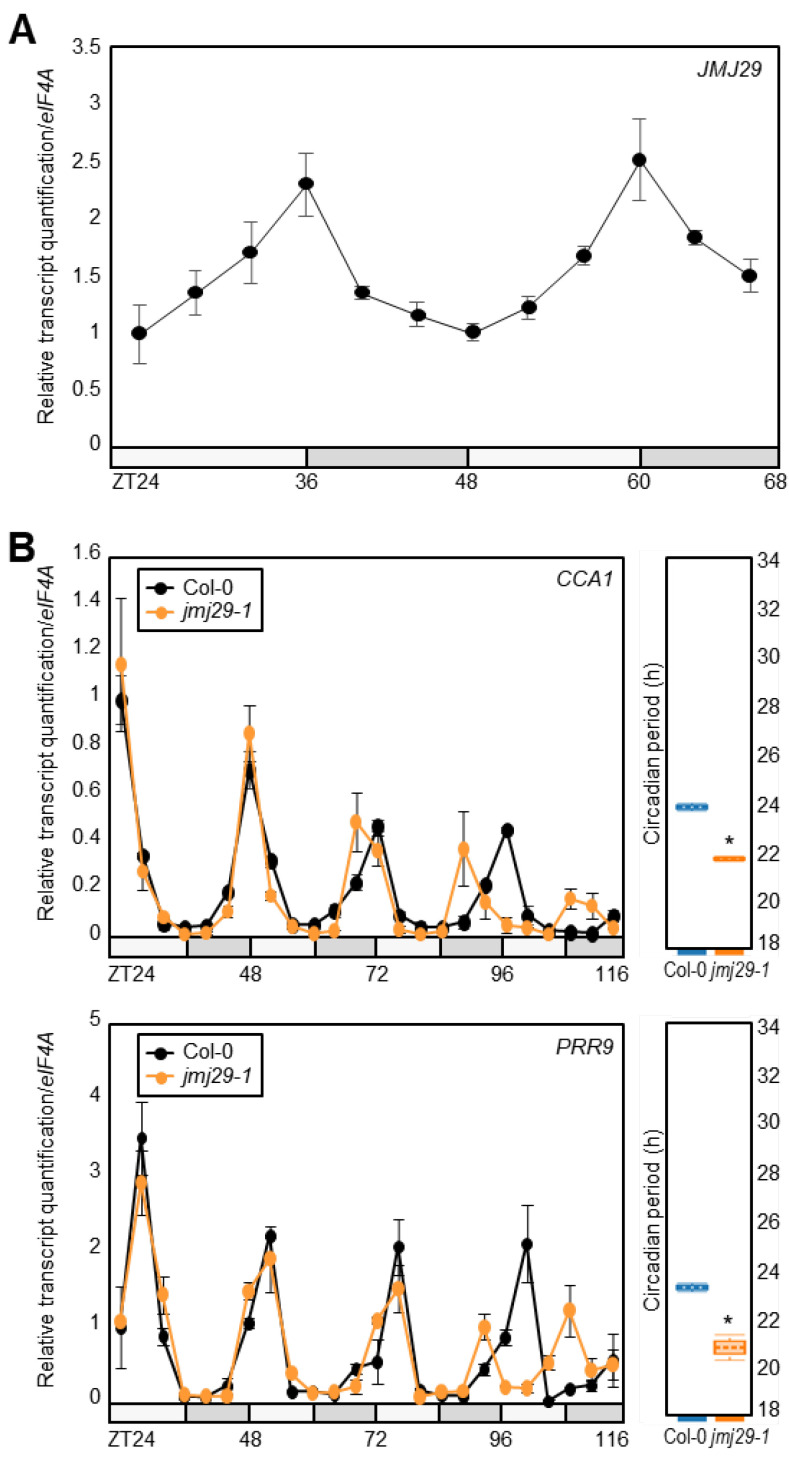
Circadian regulation of *JMJ29.* (**A**) Analysis of *JMJ29* circadian expression. Seedlings grown under neutral day conditions (ND) for 14 days were transferred to continuous light conditions (LL) at Zeitgeber Time 0 (ZT0). (**B**) Altered circadian oscillation in *jmj29-1*. Two-week-old seedlings grown under ND were transferred to LL at ZT0. Whole seedlings (*n* > 15) were harvested from ZT24 to ZT116 to analyze transcript levels of *CCA1* and *PRR9*. Period estimates were calculated using FFT-NLLS (Biodare2). Statistical significance was determined by Student’s *t*-test (* *p* < 0.05). In (**A**) and (**B**), two independent experiments were averaged. Gene expression values were normalized relative to *eIF4A* expression and represented as *n*-fold relative to the value of the ZT24. Bars indicate the standard error of the mean. The white and gray boxes indicate the subjective day and night, respectively.

Accumulating evidence has supported that histone demethylase activity is important for the rhythmic oscillation of histone methylation at the core clock promoters [16]. Although several JMJ proteins have been characterized as crucial regulators of circadian oscillation [20,21,28], we wanted to identify additional catalytic enzymes responsible for the H3K4me3 dynamics. Among others, we noticed the *JMJ29* gene, which encodes the JHDM2/KDM3 group Jumonji-C domain-containing histone demethylase [22,29], because *JMJ29* expression displayed circadian rhythm with a peak at dusk (Figure 1A) [30].

We then obtained a *JMJ29*-deficient *jmj29-1* mutant [22] and analyzed rhythmic expression of the core clock oscillator genes, *CCA1* and *PRR9* (Figure 1B). Quantitative real-time RT-PCR (RT-qPCR) analysis revealed that *jmj29-1* mutation led to alterations in circadian oscillation. Circadian period of clock gene expression was shortened in *jmj29-1* mutants, compared with wild type (Figure 1B). Consistently, expression of a circadian output gene, *COLD, CIRCADIAN RHYTHM, AND RNA BINDING* (*CCR2*), also displayed advanced rhythmic phase, possibly due to a period shortening (Supplementary Figure S1). These results suggest that JMJ29 is involved in sustaining circadian oscillation under free-running conditions.

### 3.2. JMJ29 Binds to the CCA1 and PRR9 Promoters

**Figure 2:**
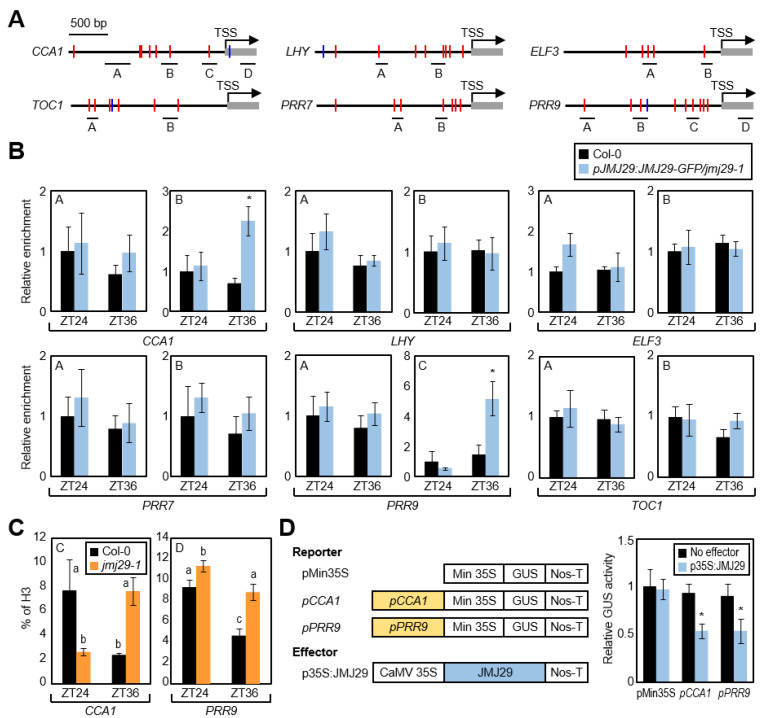
Binding of JMJ29 to the *CCA1* and *PRR9* promoters. (**A**) Promoter analysis of core clock genes. Underbars represent the regions of PCR amplification after chromatin immunoprecipitation (ChIP). E-boxes and LBSs are represented by red and blue lines, respectively. (**B**) Binding of JMJ29 to clock gene promoters. Two-week-old seedlings grown under ND condition were subjected to LL at ZT0. Plants were harvested at ZT24 and ZT36 for ChIP with anti-GFP antibody, and statistical significance of the measurements was analyzed with Student’s *t*-test (* *p* < 0.05; difference between Col-0 and *pJMJ29:JMJ29-GFP/jmj29-1* transgenic plants). (**C**) H3K4me3 levels at the *CCA1* and *PRR9* loci in *jmj29-1* mutant. Two-week-old seedlings grown in ND condition were subjected to LL at ZT0. Plants were harvested at ZT24 and ZT36 for ChIP with anti-H3 and anti-H3K4me3 antibodies, and H3K4me3 levels were normalized to total histone H3 protein levels. Statistical significance of the measurements was analyzed by one-way ANOVA with Fisher’s *post hoc* test, * *p* < 0.05. (**D**) Transient expression assays. The recombinant reporter and effector constructs were co-expressed transiently in *Arabidopsis* protoplasts. The GUS activity was determined fluorometrically. Statistical significance of the measurements was determined by Student’s *t*-test (* *p* < 0.05). In (**B**), (**C**)**,** and (**D**), biological triplicates were averaged. Bars indicate the standard error of the mean. Min 35S, minimal 35S promoter; CaMV, Cauliflower mosaic virus; Nos-T, nopaline synthase terminator.

We then asked whether JMJ29 regulates circadian clock activity through direct binding to the core clock genes. To this end, we performed chromatin immunoprecipitation (ChIP) assays using the *pJMJ29:JMJ29-GFP/jmj29-1* transgenic plants [16,20,21]. ChIP enrichment analysis in the promoter regions of core clock genes, which include key clock-related *cis*-elements, such as E-boxes and LUX-binding sites (LBSs) [8,9] (Figure 2A), showed that JMJ29 was associated specifically at the *CCA1* and *PRR9* promoters (Figure 2B and Supplementary Figure S2), and none of the other examined regions were targeted by JMJ29 (Figure 2B). We further explored whether JMJ29 rhythmically bound to the *CCA1* and *PRR9* promoters. The JMJ29 protein accumulation was likely to be correlated with the oscillation pattern of *JMJ29* transcripts (Figure 1A), and consistently, binding of JMJ29 to the *CCA1* and *PRR9* loci was observed preferentially at dusk (Figure 2B), when expression of the two morning genes was low [2,3,14].

JMJ29 that belongs to the JHDM2/KDM3 group is known to primarily catalyze H3K9 demethylation [22]. However, circadian expression of *CCA1* and *PRR9* is known to be independent of H3K9me1/2 levels [12]. Further, JMJ29 did not significantly influence H3K9me2 accumulation at the *CCA1* and *PRR9* loci (Supplementary Figure S3). Thus, we suspected that JMJ29 might contribute to changes in H3K4me3 accumulation locally at the *CCA1* and *PRR9* promoters. ChIP assays with an anti-H3K4me3 antibody showed that, in wild-type seedlings, reduction of H3K4me3 accumulation at the *CCA1* and *PRR9* loci occurred around dusk (Figure 2C) when *JMJ29* was actively expressed (Figure 1A). However, the reduction of H3K4me3 levels at dusk was diminished in the *jmj29-1* mutant (Figure 2C). Although histone modification at the *CCA1* and *PRR9* loci was altered in *jmj29-1*, their expression was not immediately changed (Figure 1B), possibly due to extensive transcriptional feedback loops. Thus, the JMJ29 protein promotes H3K4me3 demethylation at the *CCA1* and *PRR9* loci and subsequently influences their expression at later stages of free running.

Since H3K4me3 is responsible for gene activation [16,31], JMJ29 most likely inhibits the expression of *CCA1* and *PRR9*. To test this hypothesis, we carried out transient expression assays using *Arabidopsis* protoplasts. The *CCA1* and *PRR9* promoters were fused to the 35S minimal promoter in the reporter plasmid. The recombinant reporter plasmid and the effector construct expressing the *JMJ29* gene were co-transfected into *Arabidopsis* protoplasts (Figure 2D). Co-expression of these constructs repressed the GUS activity by ~50% (Figure 2D). Although regulation of *CCA1* and *PRR9* expression by JMJ29 was not immediately observed in wild-type seedlings (Figure 1B), overexpression of *JMJ29* clearly led to the repression of *CCA1* and *PRR9* (Figure 2D). These results indicate that dusk-expressed JMJ29 is recruited to the *CCA1* and *PRR9* promoters and represses expression by promoting H3K4me3 demethylation.

### 3.3. Protein–Protein Interaction between JMJ29 and ELF3

**Figure 3:**
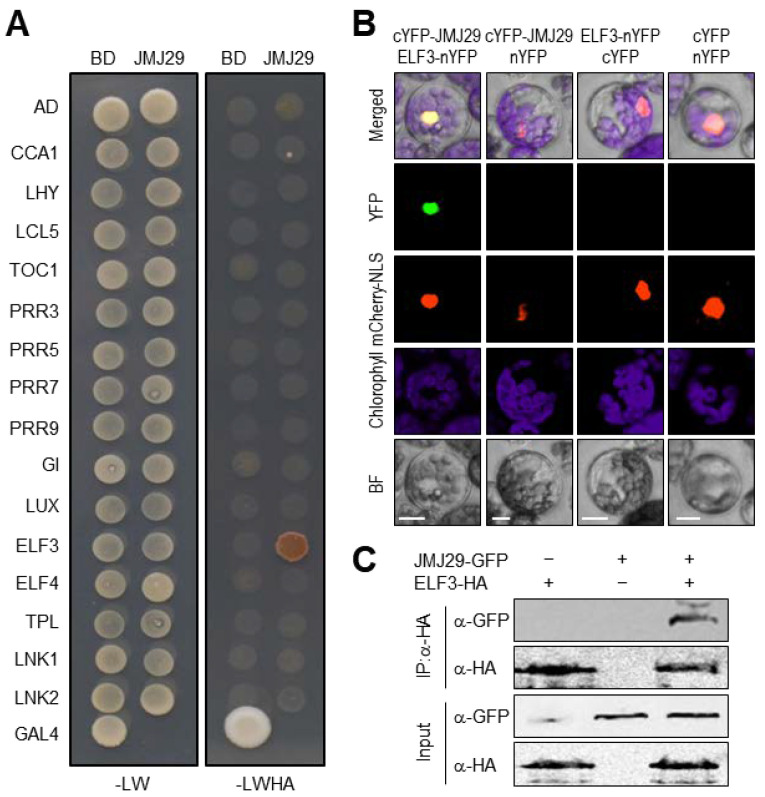
Interaction of ELF3 with JMJ29. (**A**) Yeast-two-hybrid (Y2H) assays. Y2H assays were performed with the JMJ29 protein fused to the GAL4 BD, and clock proteins fused to the GAL4 AD. -LWHA represents Leu, Trp, His, and Ade drop-out plates. -LW represents Leu and Trp drop-out plates. GAL4 was used as a positive control. The experiments were repeated three times with similar results, and one representative experiment is shown. (**B**) Bimolecular fluorescence complementation (BiFC) assays. Partial YFP fragment fusion constructs containing either JMJ29 or ELF3 were transiently co-expressed in *Arabidopsis* protoplasts. The mCherry-NLS construct was used as a positive control of nuclear localization. The number of protoplast cells yielding detectable fluorescent signals was counted, and the representative image is shown only when we observed positive interactions over negative controls. Scale bars = 20 μm. (**C**) Coimmunoprecipitation (Co-IP) assays. *35S:ELF3-HA* and *35S:JMJ29-GFP* constructs are transiently co-expressed in *Arabidopsis* protoplasts. Epitope-tagged proteins were detected immunologically using corresponding antibodies. The experiments were repeated three times with similar results, and one representative experiment is shown. IP, immunoprecipitation.

Given that JMJ29 has no selective DNA-binding domain responsible for the recognition of specific *cis*-elements like other JMJ proteins [22], we hypothesized that additional molecular component(s) were required for JMJ29 regulation of *CCA1* and *PRR9* expression. To examine this hypothesis, we conducted yeast-two-hybrid assays and tested whether JMJ29 interacts with core clock proteins. The JMJ29-GAL4 DNA BD fusion construct was co-expressed with a construct expressing a core clock protein fused to GAL4 AD in yeast cells. As a result, JMJ29 specifically interacted with ELF3 (Figure 3A), but none of the other clock proteins were associated (Figure 3A).

To support the *in vivo* interaction of JMJ29 with ELF3, we conducted bimolecular fluorescence complementation (BiFC) analysis in *Arabidopsis* protoplasts. The *JMJ29* gene was fused to the sequence encoding the C-terminal half of YFP (cYFP), and the *ELF3* gene was fused to the sequence encoding the N-terminal half of YFP (nYFP). The fusion constructs were then transiently co-expressed in *Arabidopsis* protoplasts. Co-expression of the JMJ29-ELF3 combination generated yellow fluorescence exclusively in the nucleus (Figure 3B), while expression with empty vectors did not show visible fluorescence (Figure 3B). Consistent with the fact that ELF3 is a component of the EC [32,33], JMJ29 also physically associated with other EC components, ELF4 and LUX (Supplementary Figure S4). The discrepancy of interactions between JMJ29 and EC components in yeast and plant cells might be due to the requirement of plant-specific molecular factors in the protein-protein interactions. We also confirmed *in planta* interactions of JMJ29 and ELF3 (Figure 3C). These results indicated that JMJ29 interacts with the EC to directly repress *CCA1* and *PRR9*.

### 3.4. The EC Facilitates Transcriptional Repression of CCA1 and PRR9

**Figure 4:**
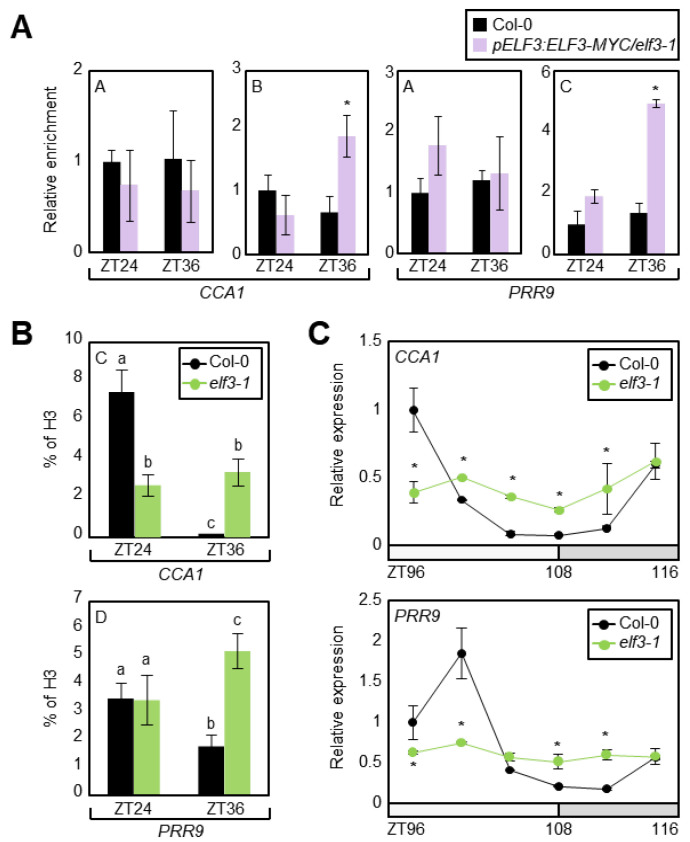
Repression of *CCA1* and *PRR9* expression by EC via H3K4me3 demethylation. (**A**) Binding of ELF3 to the *CCA1* and *PRR9* promoters. Two-week-old plants grown in ND condition were subjected to LL at ZT0. Plants were harvested at ZT24 and ZT36 for ChIP assays. Enrichment of target chromatin regions was analyzed by ChIP-qPCR with the primer sets shown in Figure 2A. Biological triplicates were averaged and statistically analyzed with Student’s *t*-test (* *p* < 0.05). (**B**) H3K4me3 levels at the *CCA1* and *PRR9* loci in *elf3-1* mutant. Plants were harvested at ZT24 and ZT36 for ChIP with anti-H3 and anti-H3K4me3 antibodies, and H3K4me3 levels were normalized to total histone H3 protein levels. Biological triplicates were averaged, and statistical significance of the measurements was analyzed by one-way ANOVA with Fisher’s *post hoc* test (*p* < 0.05). (**C**) Transcript levels of *CCA1* and *PRR9* in *elf3-1* mutant. Two-week-old seedlings grown under ND were transferred to LL at ZT0. Whole seedlings (*n* > 15) were harvested from ZT96 to ZT116. Gene expression values were normalized to the *eIF4A* expression. Two technical replicates were averaged and statistically analyzed with Student’s *t*-test (*p < 0.05). Bars indicate the standard error of the mean. The white and gray boxes indicate the subjective day and night.

Considering that the JMJ29 protein is involved in the temporal regulation of *CCA1* and *PRR9*, we needed to confirm whether the EC function is also relevant in rhythmic H3K4me3 accumulation at the *CCA1* and *PRR9* promoters. We first employed *pELF3:ELF3-MYC/elf3-1* transgenic plants [23] and performed ChIP assays using an anti-MYC antibody. In agreement with the temporal association of JMJ29 to the *CCA1* and *PRR9* loci (Figure 2B), ELF3 also bound to the *CCA1* and *PRR9* promoters in the regions where JMJ29 was recruited, preferentially at dusk (Figure 2A, 2B, and Figure 4A).

Since the EC recruits JMJ29 to repress gene expression via H3K4me3 demethylation, we analyzed H3K4me3 levels at the *CCA1* and *PRR9* loci in the *elf3-1* mutant. ChIP-qPCR analysis for H3K4me3 accumulation revealed that the reduction of H3K4me3 levels at the *CCA1* and *PRR9* loci around dusk was impaired in *elf3-1* (Figure 4B), similar to that in *jmj29-1* (Figure 2C). We also examined expression of *CCA1* and *PRR9* in *elf3-1* and found that their expression was arrhythmic and increased around dusk (Figure 4C), when JMJ29 and EC were active (Figure 1A) [33]. The expression amplitude of *CCA1* and *PRR9* in *elf3-1* was a bit different from previous reports [8,34], probably because of our growth conditions.

The transcriptional repression of *CCA1* and *PRR9* by EC was also confirmed by transient expression assays (Supplementary Figure S5). The recombinant reporter plasmid containing the *CCA1* or *PRR9* promoter and the effector construct expressing *ELF3*, *ELF4*, or *LUX* were co-transfected into *Arabidopsis* protoplasts. GUS activity measurement showed that the EC components could bind to the *CCA1* and *PRR9* promoters and repressed expression (Supplementary Figure S5). These results indicate that the EC is responsible for the H3K4me3-dependent regulation of *CCA1* and *PRR9*.

### 3.5. The EC and JMJ29 Are Interdependent in Regulating CCA1 and PRR9 Expression

**Figure 5:**
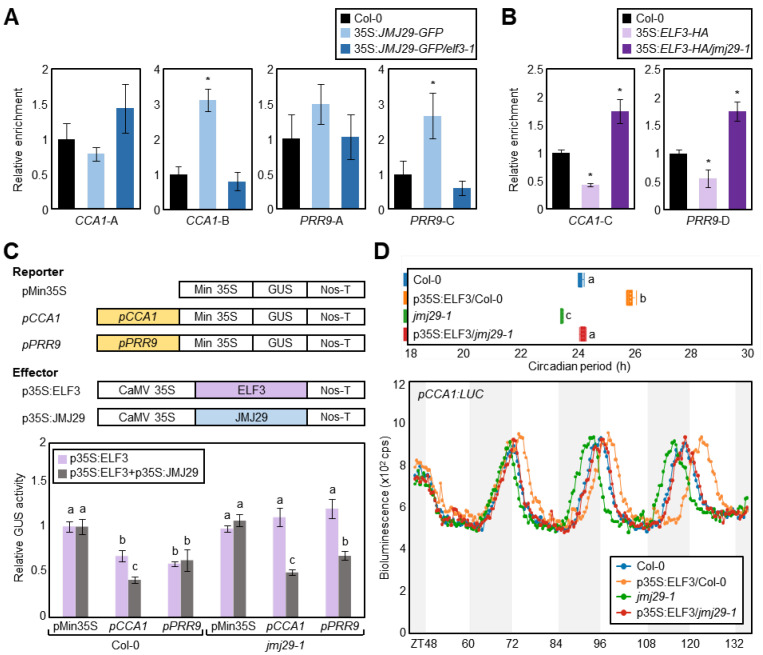
Interdependency of EC and JMJ29 in repression of *CCA1* and *PRR9*. (**A**) Binding of JMJ29 to the *CCA1* and *PRR9* loci in *elf3-1*. *35S:JMJ29-GFP* construct was transfected in protoplasts isolated from two-week-old seedlings grown under NDs. The protoplasts were harvested at ZT36 for ChIP using anti-GFP antibody. (**B**) H3K4me3 levels in *35S:ELF3-HA/jmj29-1*. 35S:ELF3-HA construct was transfected in protoplasts isolated from two-week-old seedlings grown under NDs. The protoplasts were harvested at ZT36 for ChIP using anti-H3K4me3 antibody. In (**A**) and (**B**), the indicated genomic regions (see Figure 2A) were analyzed by ChIP-qPCR. Biological triplicates were averaged and statistically analyzed with Student’s *t*-test (* *p* < 0.05). Bars indicate the standard error of the mean. (**C**) Transient expression assays using *Arabidopsis* protoplasts. The recombinant reporter and effector constructs were co-expressed transiently in *Arabidopsis* protoplasts, and then GUS activity was determined fluorometrically. Biological triplicates were averaged, and statistical significance of the measurements was analyzed by one-way ANOVA with Fisher’s *post hoc* test (*p* < 0.05). Bars indicate the standard error of the mean. Min 35S, minimal 35S promoter; CaMV, Cauliflower mosaic virus; Nos-T, nopaline synthase terminator. (**D**) Impact of *ELF3* overexpression in circadian period. The *35S:ELF3* effector plasmid was transfected into wild-type and *jmj29-1* mutant protoplasts. Biological triplicates were averaged, and statistical significance of the measurements was analyzed by one-way ANOVA (*p* < 0.05). Bars indicate the standard error of the mean.

Our results demonstrated that the EC and JMJ29 act together in the repression of *CCA1* and *PRR9*. We thus asked whether ELF3 repression of *CCA1* and *PRR9* required JMJ29. The importance of EC function for JMJ29 binding to the *CCA1* and *PRR9* promoters was tested using wild-type and *elf3-1* mutant protoplasts transiently expressing *35S:JMJ29-GFP.* ChIP analysis with the anti-GFP antibody showed that JMJ29 binding to the *CCA1* and *PRR9* loci was reduced in the *elf3-1* mutant background compared with the wild-type background (Figure 5A). Since JMJ29 protein catalyzes H3K4me3 demethylation at the cognate regions (Figure 2C), H3K4me3 levels at the *CCA1* and *PRR9* loci were also measured in wild-type and *jmj29-1* protoplasts transiently expressing *35S:ELF3-HA*. H3K4me3 levels were reduced by overexpression of *ELF3* in wild-type protoplasts, but ELF3-induced H3K4me3 demethylation was compromised in the *jmj29-1* mutant protoplasts (Figure 5B).

We also performed transient gene expression assays using *Arabidopsis* protoplasts. Reporter GUS activity measurement indicated that ELF3 significantly repressed *CCA1* and *PRR9* promoter activities in wild-type protoplasts, but the ELF3 function disappeared in the *jmj29-1* mutant (Figure 5C). The impaired ELF3 function was restored by complementation of JMJ29 (Figure 5C). In further support, overexpression of *ELF3* led to prolonged circadian period in wild-type protoplasts, but the ELF3 function was impaired in *jmj29-1* mutant protoplasts (Figure 5D). These results demonstrate that both EC and JMJ29 are simultaneously involved in repressing *CCA1* and *PRR9* expression in circadian cycles.

**Figure 6:**
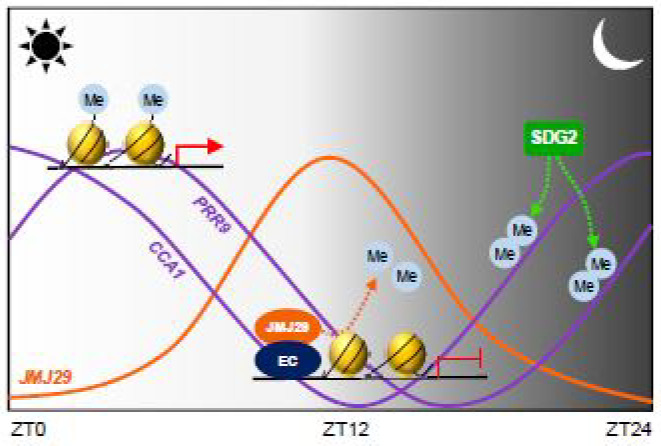
Proposed working model of the EC-JMJ29 complex in circadian control. Circadian expression of *CCA1* and *PRR9* are associated with H3K4me3 levels. SDG2 possibly catalyzes the H3K4me3 deposition at the *CCA1* and *PRR9* promoters at dawn, facilitating raising phase of gene expression. After peak expression, the declining phase of *CCA1* and *PRR9* is shaped by the Evening Complex (EC) and JMJ29. The EC recruits JMJ29 to the *CCA1* and *PRR9* promoters and removes H3K4me3 at dusk, maintaining low levels of expression during evening time.

In summary, the *CCA1* and *PRR9* genes are regulated by rhythmic changes in H3K4me3 levels. SDG2 is possibly responsible for the raising phase of their expression [20]. After peak expression, JMJ29 is recruited via the interaction with ELF3, a component of the EC. The EC-JMJ29 induces H3K4me3 demethylation at the *CCA1* and *PRR9* loci to reach their basal expression during evening time (Figure 6).

## 4. Discussion

Rhythmic transcriptional regulation of core clock genes is intimately connected with chromatin status, which is under the extensive control of DNA methylation, histone modifications, and histone variant exchange [35,36,37,38]. To date, changes in histone modifications, such as H3K56ac, H3K9/14ac, and H3K4me3, at clock gene promoters are known to be particularly important for circadian dynamics in *Arabidopsis* [13,16]. Notably, the circadian accumulation of H3K4me3 at core clock gene loci is mediated by antagonistic actions of histone methyltransferases and histone demethylases. The SDG2/ATXR3 histone methyltransferase deposits H3K4me3 globally at core clock gene loci [20]. After peak expression, multiple histone demethylases, which belong to the KDM1/LSD1 or JmjC family [39], regulate a specific subset of clock genes, returning to the basal expression level of their target genes [39]. For example, LDL1, LDL2, JMJ14, and JMJ30 allow the diurnal repression of *CCA1* and *LHY*, possibly through controlling rhythmic H3K4me3 accumulation [20,21,39]. Here, we show that JMJ29 regulates circadian oscillations by repressing *CCA1* and *PRR9* expression. JMJ29 interacts with the EC component ELF3, and the EC rhythmically recruits JMJ29 to the *CCA1* and *PRR9* promoters at dusk, when EC and JMJ29 are active. Then, the JMJ29 protein catalyzes H3K4me3 demethylation to maintain low levels of *CCA1* and *PRR9* expression during evening time.

However, several questions remain to be elucidated. Although JMJ29 is a member of the JHDM2/KDM3 group JmjC domain-containing histone demethylases [29], which were identified to predominantly catalyze the H3K9me1/2 demethylation in eukaryotes [40,41,42,43], our results showed that JMJ29 was also able to remove H3K4me3, especially at core clock gene loci. JMJ29 most likely has a pervasive role in H3K9me1/2 demethylation to activate gene expression, but the JMJ29 protein is also relevant in transcriptional repression locally at certain chromatin regions via H3K4me3 demethylation. It is currently unclear what structural and biochemical features of JMJ29 enable it to have the dual functions. It is also a reasonable question, to determine which catalytic activity of JMJ29 is predominant at a given sequence context. Additionally, even though EC has a broader spectrum of clock gene binding, binding of JMJ29 is limited to the *CCA1* and *PRR9* promoters. EC also interacts with various chromatin modifiers and remodelers, in addition to JMJ29. While H3K4me3 demethylation at the core clock genes by ELF3 was dependent on JMJ29, regulation of circadian gene expression by ELF3 was further elaborated by additional epigenetic and molecular factors [17,44,45]. Thus, detailed molecular mechanisms should be elucidated to give a comprehensive view of circadian-chromatin networks.

In addition to circadian oscillation, the JMJ29 protein was also implicated in plant growth and development. For instance, JMJ29 participates in trichrome development. JMJ29 directly binds to the *GLABRA 3* (*GL3*) promoter and activates gene expression via H3K9me2 demethylation [22]. JMJ29 may also indirectly affect expression of *GL1* and *GL2* [22]. Consistently, the trichome density on leaves and inflorescent stems were reduced in *jmj29* mutants with a low expression of *GL1*, *GL2*, and *GL3* [22]. Based on our study, it is likely that trichome development might be under the circadian control, and JMJ29 relays diurnal cues to induce proper developmental processes. Altogether, the JMJ29 protein is likely to integrate temporal information into circadian oscillation programs and also a variety of output processes optimizing plant growth and development.

## 5. Conclusions

Diurnal changes in H3K4me3 levels shape the rhythmic oscillation of core clock gene expression. The global deposition of H3K4me3 at the clock gene loci is known to be catalyzed by SDG2/ATXR3. The removal of H3K4me3 at core clock genes is mediated by multiple histone demethylases, each of which has a limited target gene spectrum. The JMJ29 protein interacts with the EC, and the EC-JMJ29 complex is able to associate the *CCA1* and *PRR9* promoters at dusk. The JMJ29 protein promotes H3K4me3 demethylation and maintains their expression at low levels during evening time.

## Data Availability

The data presented in this study are available on request from the corresponding author.

## Supplementary Materials

The following are available online at https://www.mdpi.com/article/10.3390/genes12040529/s1, Figure S1: Circadian expression of *CCR2* in *jmj29-1*. Figure S2: Binding of JMJ29 to coding regions of *CCA1* and *PRR9*. Figure S3: H3K9me2 accumulation at the *CCA1* and *PRR9* loci. Figure S4: Interaction of JMJ29 with ELF4 and LUX. Figure S5: Transient expression assays using *Arabidopsis* protoplasts. Table S1: List of primers used in this study. Table S2: List of primers used in chromatin immunoprecipitation (ChIP) assays.
