# Supplementary material for: The Arabidopsis JMJ29 Protein Controls Circadian Oscillation through Diurnal Histone Demethylation at the CCA1 and PRR9 Loci
Source: Genes (Basel). 2021 Apr 5;12(4):529. doi: 10.3390/genes12040529 (PMC8066055; doi:10.3390/genes12040529)
Supplement: Supplementary file 1 [file genes-12-00529-s001.pdf]

Supplementary Figures

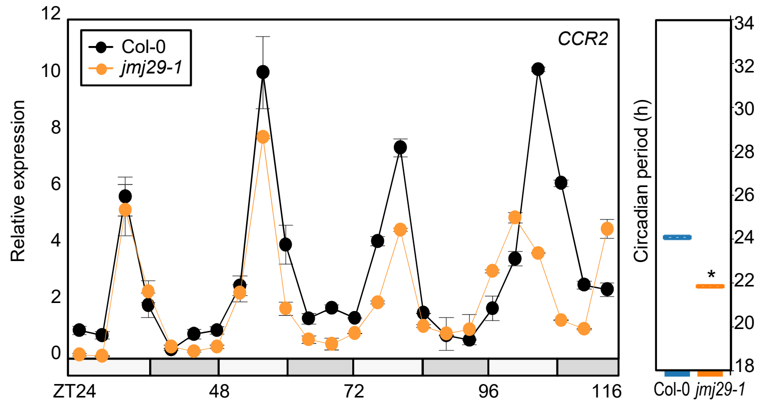

Supplementary Figure S1. Circadian expression of *CCR2* in *jmj29-1*
Two-week-old seedlings grown under neutral day (ND) conditions were transferred to continuous light (LL) conditions at ZT0. Whole seedlings were harvested from ZT24 to ZT116 to analyze transcript accumulation. Two technical replicates were averaged, and period estimates were calculated using FFT-NLLS (Biodare2). Bars indicate the standard error of the mean. The white and grey boxes indicate the subjective day and night, respectively.

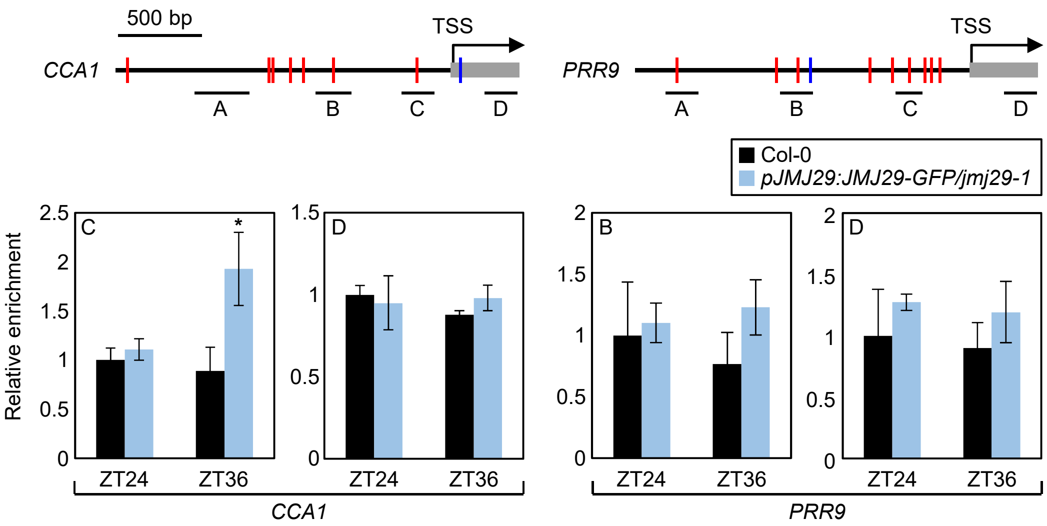

Supplementary Figure S2. Binding of JMJ29 to coding regions of *CCA1* and *PRR9*

Two-week-old plants entrained with ND cycles were subjected to LL at ZT0. Plants were harvested at ZT24 and ZT36 for ChIP analysis with anti-GFP antibody. Enrichment of fragmented genomic regions was analyzed by ChIP-qPCR. Biological triplicates were averaged, and statistical significance was determined by Student's *t*-test (**P* < 0.05). Bars indicate standard error of the mean. TSS, transcription start site.

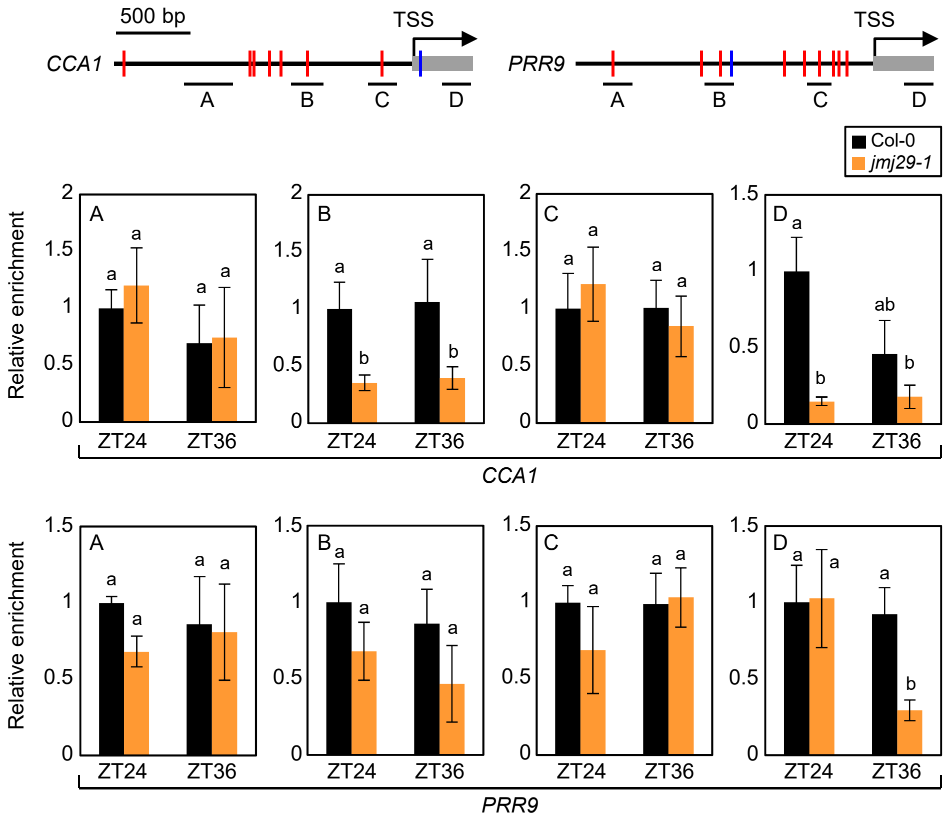

Supplementary Figure S3. H3K9me2 accumulation at the *CCA1* and *PRR9* loci

Two-week-old plants entrained with ND cycles were subjected to LL at ZT0. Plants were harvested at ZT24 and ZT36 for ChIP analysis with anti-H3K9me2 antibody. Enrichment of fragmented genomic regions was analyzed by ChIP-qPCR. Different letters represent a significant difference at $P < 0.05$ (one-way ANOVA with Fisher's *post hoc* test). Biological triplicates were averaged. Bars indicate the standard error of the mean. TSS, transcription start site.

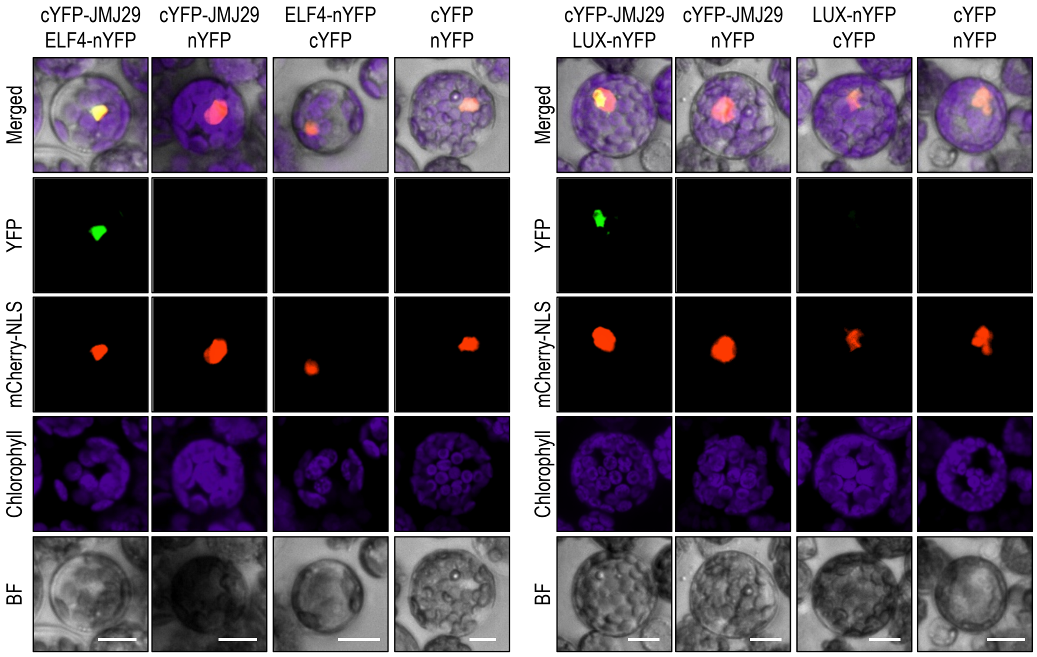

Supplementary Figure S4. Interaction of JMJ29 with ELF4 and LUX

Constructs expressing JMJ29, ELF4 and LUX fused either to the N-terminus or C-terminus fragment of YFP were co-transfected into *Arabidopsis* protoplast cells. Scale bars = 20 µm. BF, bright field.

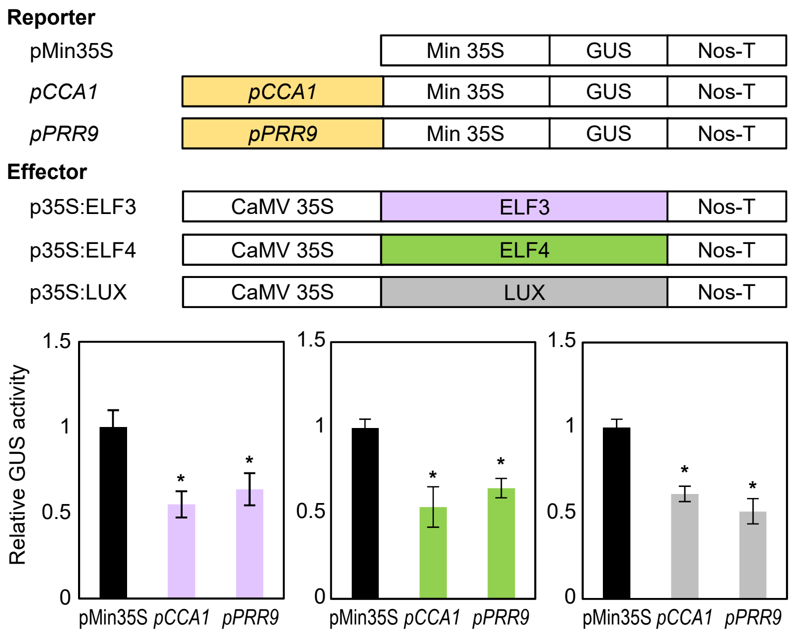

Supplementary Figure S5. Transient expression assays using *Arabidopsis* protoplasts

The recombinant reporter and effector constructs were co-expressed transiently in *Arabidopsis* protoplasts, and GUS activity was determined. Luciferase gene expression was used to normalize GUS activity. The normalized values in control protoplasts were set to 1 and represented as relative activation. Biological triplicates were averaged, and statistical significance was determined by Student's *t*-test (*P < 0.05). Bars indicate the standard error of the mean.

Supplementary Tables

Supplementary Table S1. List of primers used in this study

Primer	Usage	Sequence
eIF4a-F	RT-qPCR	5′-TGACCACACAGTCTCTGCAA
eIF4a-R	RT-qPCR	5′-ACCAGGGAGACTTGTTGGAC
JMJ29-F	RT-qPCR	5′-GATCGAGCCATGGACATTTG
JMJ29-R	RT-qPCR	5′-GCGACTTTTGTGCACGACTT
CCA1-F	RT-qPCR	5′-GATCTGGTTATTAAGACTCGGAAGCCATATAC
CCA1-R	RT-qPCR	5′-GCCTCTTTCTCTACCTTGGAGA
PRR9-F	RT-qPCR	5′-TTGGTCCTGAGCTTGGACTTT
PRR9-R	RT-qPCR	5′-GCTTACGCTTGATGATCCGA
CCR2-F	RT-qPCR	5′-CGTTATTGATTCCAAGATCA
CCR2-R	RT-qPCR	5′-ATCCTTCATGGCTTTCTCAT
pCCA1-F(pMin35S)	cloning	5′-GAGGATCCGAACTTGTAGGCATCGGTTACAC
pCCA1-R(pMin35S)	cloning	5′-GAAAGCTTCACTAAGCTCCTCTACACAACTT
pPRR9-F(pMin35S)	cloning	5′-GAGGATCCCGCGGCCACTAACGAAATTTG
pPRR9-R(pMin35S)	cloning	5′-GAAAGCTTCACTAAGCTCCTCTACACAACTT
35S:JMJ29-GFP-F	cloning	5′-GAGTCGACATGGATTCTGGAGTTAAATTGGAG
35S:JMJ29-GFP-R	cloning	5′-GACCCGGGCAAGAGATAAAAGACTTGCCTCGAG
35S:ELF3-HA-F	cloning	5′-CACAAGTTTGTACAAAAAAGCTGAAATGAAGAGAGGGAAAGATGAGG
35S:ELF3-HA-R	cloning	5′-GGCACCACTTTGTACAAGAAATTAAGGCTTAGAGGAGTCATAGC
JMJ29-F(pGBKT7)	cloning	5′-GACCCGGGGATGGATTCTGGAGTTAAATTGG
JMJ29-R(pGBKT7)	cloning	5′-GACTGCAGTCAAAGAGATAAAAGACTTGCCTC
CCA1-F(pGADT7)	cloning	5′-GAGCCGGCATGGAGACAAATTCGTCTGG
CCA1-R(pGADT7)	cloning	5′-GAGAATTCTCATGTGGAAGCTTGAGTTTC
LHY-F(pGADT7)	cloning	5′-GACATATGATGGATACTAATACATCTGGAGAAGAATTATTAG
LHY-R(pGADT7)	cloning	5′-GAGGATCCTCATGTAGAAGCTTCTCCTTCC
LCL5-F(pGADT7)	cloning	5′-GACCATGGAGATGAGCTCGTCGCCGTC
LCL5-R(pGADT7)	cloning	5′-GAGAATTCTTATGCTGATTTGTCGCTTGTTG
TOC1-F(pGADT7)	cloning	5′-GACCATGGAGATGGATTTGAACGGTGAGTG
TOC1-R(pGADT7)	cloning	5′-GACCCGGGTCAAGTTCCCAAAGCATCATC
PRR3-F(pGADT7)	cloning	5′-GACCATGGAGATGTGTTTTAATAACATTGAAACTGG
PRR3-R(pGADT7)	cloning	5′-GAGGATCCTCAATTGTCTTCACTTCCTGATTTATG
PRR5-F(pGADT7)	cloning	5′-GACATATGATGTGGCAAACGTGGC
PRR7-F(pGADT7)	cloning	5′-GACCATGGATATGAATGCTAATGAGGAGGGG
PRR7-R(pGADT7)	cloning	5′-GACCCGGGTTAGCTATCCTCAATGTTTTTTATGTC
PRR9-F(pGADT7)	cloning	5′-GACCATGGATATGGGGGAGATTGTGGTTTTAAG
PRR9-R(pGADT7)	cloning	5′-GACCCGGGTCATGATTTTGTAGACGCGTCTG
GI-F(pGBKT7)	cloning	5′-GAGAATTCATGGCTAGTTCATCTTCATCTGAGAG
GI-R(pGBKT7)	cloning	5′-GAGGATCCCTTATTGGGACAAGGATATAGTACAGCC
LUX-F(pGADT7)	cloning	5′-GACCATGGATATGGGAGAGGAAGTACAAATGAG
LUX-R(pGADT7)	cloning	5′-GACCCGGGCTACATGATACTTTGTATGATCCTCTCC
ELF3-F(pGADT7)	cloning	5′-GAGGATCCATGAAGAGAGGGAAAGATGAGG
ELF3-R(pGADT7)	cloning	5′-GACTCGAGTTAAGGCTTAGAGGAGTCATAGCG
ELF4-F(pGADT7)	cloning	5′-GACCATGGAGATGAAGAGGAACGGCGAG
ELF4-R(pGADT7)	cloning	5′-GAGAATTCTTAAGCTCTAGTTCCGGCAG
TPL-F(pGADT7)	cloning	5′-GACCATGGAGATGTCTTCTCTTAGTAGAGAGCTCG
TPL-R(pGADT7)	cloning	5′-GACCCGGGTCAAACAGGTGACGCCGTTGGTTG
LNK1-F(pGADT7)	cloning	5′-GAGAATTCATGTCGGACTTGTACATTCATGAG
LNK1-R(pGADT7)	cloning	5′-GACTCGAGTTAATTGTTGTCACTTGTTACAACTTCTG
LNK2-F(pGADT7)	cloning	5′-GACCATGGAGATGATATGGGGTGATGATGCTG
LNK2-R(pGADT7)	cloning	5′-GACCCGGGTCACAATTTTCTTTTGTTTCCTTG

RT-qPCR primers were designed using the Primer Express Software installed into the Applied Biosystems PCR System. The sizes of PCR products ranged from 80 to 300 nucleotides in length. F, forward primer; R, reverse primer.

Supplementary Table S2. List of primers used in chromatin immunoprecipitation (ChIP) assays

Primer	Sequence
CCA1(A)-F	CTTCTCTTTGTATCACTTGAACCAA
CCA1(A)-R	GAATTTGAGTCTTCCATTCTCAGTATTA
CCA1(B)-F	ATATAAAACTATGGCCCAAATAAGTTTAG
CCA1(B)-R	ATCTTGATCTAGTGGGACCTAC
CCA1(C)-F	CATTTCCGTAGCTTCTGGTCTCTT
CCA1(C)-R	ATCAGCTTGGATTCGATAAAGATTC
CCA1(D)-F	ACTCGGAAGCCATATACGATAAC
CCA1(D)-R	CAAAGCTTCAATGAATCTATTATG
LHY(A)-F	CTACATGCTTCGGTTAAGAC
LHY(A)-R	TCTTCATCTTTTCATATAATATCATGCAATG
LHY(B)-F	TCCTCCATGGCTACTCTCAAGG
LHY(B)-R	TCAGCAGCCAAACAGAGATCTTAG
ELF3(A)-F	TTTAGTAAATAAGAGTGTCCAAGTG
ELF3(A)-R	AGAAACATAGCAAAAGCTCTAG
ELF3(B)-F	AACCTCTAACATGGTAATATATCTATG
ELF3(B)-R	ATCATCCAATACATCACTTTTTG
TOC1(A)-F	AAGAAACTATCCGAATAACTTCATGC
TOC1(A)-R	TTTGATGAAATTCCTCAGAGAAGATG
TOC1(B)-F	AACAGAAAAATAAAATTCTGATAATAG
TOC1(B)-R	AAACCAAATTTTAGGATTCG
PRR7(A)-F	TTTGTCTTTTAGCACTATACGGTC
PRR7(A)-R	TTCTCCTTCAGTGTTCCTTC
PRR7(B)-F	CTCTTCCGCCAAAATCTATTCAACGGTC
PRR7(B)-R	GAAGTTCCACGTCAGAGCGGATATTTC
PRR9(A)-F	ATCACCGTCCTCTTCAACTTC
PRR9(A)-R	TATAACTACTGTTTTTGTTGCTGTTG
PRR9(B)-F	CTTCGGATAAGCTTAAAATCATTTC
PRR9(B)-R	TCCAGGYGAAAGTGATCGATG
PRR9(C)-F	CGGCCACTAACGAAATTTGA
PRR9(C)-R	GCAGGTCCACCTTAACACGT
PRR9(D)-F	TCTCGGTAGATTAAGATCTAAAGCTCGTTG
PRR9(D)-R	CAACACTTGGTAAAACCAACAAAGCCTA

F, forward primer; R, reverse primer.
